# Glycyrrhizin-Based Hydrogels Accelerate Wound Healing of Normoglycemic and Diabetic Mouse Skin

**DOI:** 10.3390/pharmaceutics15010027

**Published:** 2022-12-21

**Authors:** Maarten A. Mees, Fleur Boone, Thomas Bouwen, Frederik Vanaerschot, Charlotte Titeca, Hanna-Kaisa Vikkula, Leen Catrysse, Anja Vananroye, Erin Koos, Stelios Alexandris, Sabine Rosenfeldt, Samuel Eyley, Joachim Koetz, Geert van Loo, Wim Thielemans, Esther Hoste

**Affiliations:** 1 Sustainable Materials Lab, Department of Chemical Engineering, Campus Kulak Kortrijk, KU Leuven, Etienne Sabbelaan 53, 8500 Kortrijk, Belgium; 2 VIB Center for Inflammation Research, Technologiepark 71, 9052 Ghent, Belgium; 3 Department of Biomedical Molecular Biology, Ghent University, Technologiepark 71, 9052 Ghent, Belgium; 4 Chemical Engineering Department, Soft Matter, Theology and Technology, KU Leuven, 3000 Leuven, Belgium; 5 Physical Chemistry and Bavarian Polymer Institute (BPI), University of Bayreuth, Universitätsstraße 30, 95447 Bayreuth, Germany; 6 Institut für Chemie, Universität Potsdam, Karl-Liebknecht-Straße 24-25, 14476 Potsdam, Germany

**Keywords:** wound healing, glycyrrhizin, hydrogel, rheology, physical hydrogel

## Abstract

Efficient wound repair is crucial for mammalian survival. Healing of skin wounds is severely hampered in diabetic patients, resulting in chronic non-healing wounds that are difficult to treat. High-mobility group box 1 (HMGB1) is an important signaling molecule that is released during wounding, thereby delaying regenerative responses in the skin. Here, we show that dissolving glycyrrhizin, a potent HMGB1 inhibitor, in water results in the formation of a hydrogel with remarkable rheological properties. We demonstrate that these glycyrrhizin-based hydrogels accelerate cutaneous wound closure in normoglycemic and diabetic mice by influencing keratinocyte migration. To facilitate topical application of glycyrrhizin hydrogels on cutaneous wounds, several concentrations of glycyrrhizinic acid in water were tested for their rheological, structural, and biological properties. By varying the concentration of glycyrrhizin, these hydrogel properties can be readily tuned, enabling customized wound care.

## 1. Introduction

Our skin provides a crucial physical and immunological barrier against environmental insults, ranging from UV radiation to wounding and microbial attacks. Skin regenerative responses are tightly orchestrated and comprise distinct overlapping phases, namely haemostasis, inflammation, proliferation, and tissue remodeling [1]. Chronic wounds, such as venous leg ulcers, pressure ulcers, and diabetic foot ulcers are characterized by defective wound repair. Next to the detrimental impact on the quality of life, these non-healing wounds represent a severe burden on global health care systems as 1 to 2% of the global population suffers from a chronic wound [2,3,4,5]. Diabetic wound treatment is challenging and currently entails negative pressure wound therapy or the application of specific wound dressings, depending on the wound characteristics (exudating, necrotic, or other) [6]. The generation of novel wound dressings for the treatment of chronic wounds is a pressing clinical need that has the potential to improve patient management and regenerative outcome.

During the inflammatory phase of wound healing, several immune cell populations, such as neutrophils, macrophages, and dendritic cells, infiltrate the site of wounding. Neutrophils can negatively impact skin regeneration by forming NETs (neutrophil extracellular traps), which are released as an anti-microbial strategy [7,8]. We previously demonstrated a key role for high-mobility group box 1 (HMGB1) in mediating neutrophil infiltration and NET formation in cutaneous wound healing [9]. HMGB1 is a pleiotropic molecule exerting different functions based on its subcellular localization. Under homeostatic conditions, HMGB1 resides in the nucleus, where it is important in regulating transcriptional activity. Upon tissue damage, HMGB1 is secreted and functions as a danger-associated molecular pattern (DAMP) can alert the immune system into action [10]. Skin injury induces a transient increase in the levels of HMGB1 in both normoglycemic and diabetic mice [11,12]. During wound healing, HMGB1 can critically mediate reparative inflammation as it regulates the infiltration of immune cells, such as neutrophils into the damaged tissue. Ablation of HMGB1 in keratinocytes, the epithelial cells of the skin, resulted in reduced infiltration of neutrophils, and reduced priming to NET formation in skin wounds leads to accelerated wound closure [9]. This suggests there could be potential therapeutic benefits for inhibition of HMGB1 in skin regeneration. Indeed, we hypothesize that inhibiting HMGB1 during wound healing might have therapeutic potential in accelerating wound repair because (a) HMGB1 functions as an important mediator of the inflammatory response upon wounding and (b) genetic deletion of HMGB1 in skin epithelial cells results in faster wound healing rates [9].

**Scheme 1:**
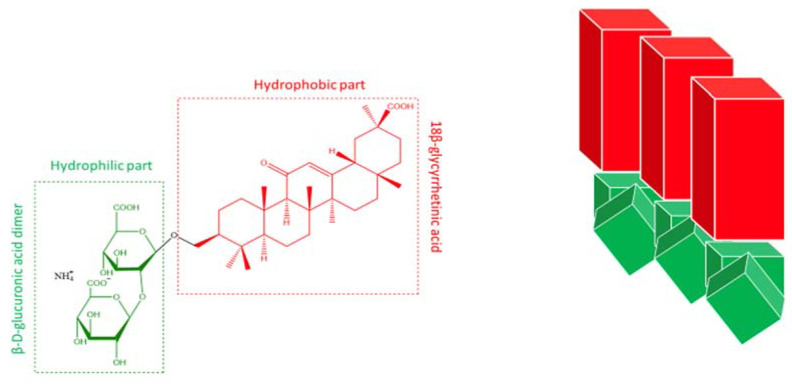
Chemical structure of β-glycyrrhizin (left) and graphical description of a GA lattice where multiple red parts (glycyrrhetinic acid) form a hydrophobic zone and the green (glucoronic acid) forms a hydrophilic zone.

A well-known inhibitor of HMGB1 is the amphiphilic molecule β-glycyrrhizin, also named glycyrrhizinic acid (GA), which is a natural triterpene glycoconjugate present in licorice roots [13,14,15]. GA is an FDA-approved food additive that is generally recognized as safe, however, excessive oral consumption can lead to hypertension and hypokalemia [16,17,18]. GA inhibits HMGB1 by direct binding through Van der Waals interactions between GA and the two functional units of HMGB1 (namely Box A and B) and interactions between the carboxyl and carbonyl groups of GA with positively charged amino acids in HMGB1 [19]. GA does not affect the nuclear function (DNA binding) of HMGB1 while the extracellular (cytokine action) function is fully inhibited by GA [19]. GA also exhibits strong anti-viral [20,21,22,23,24], anti-bacterial [25,26,27,28,29,30,31,32,33,34], and anti-inflammatory activities [35,36,37], and has been proposed as therapeutic against COVID-19 [38]. GA consists of a hydrophobic aglycon, a triterpenoid part, and a diglycon (Scheme 1), representing its hydrophilic part. Due to its amphiphilic nature, GA can form physical hydrogels by fibril formation as a result of the formation of hydrophobic zones shielded by hydrophilic glucuronic acids. The viscosity of these hydrogels depends on the GA concentration [39]. At higher temperatures, (T > 40 °C), there is a reversible formation into dynamic micelles. By lowering the temperature these micelles merge into larger constructs [40,41] or right-handed fibrils [42]. The larger fibrillar constructs are formed due to an increased intermicellar interaction [41]. The orientation of the carboxylic acid group of the triterpene moiety of GA is crucial for the formation of fibrils because α-GA forms micelles without forming hydrogels. The configuration of the steroid carboxylic acid induces different stacking at lower temperatures due to steric interference [40]. Recently efforts were made to use GA in wound healing by combining it with a polymer network [43,44].

By combining the unique capabilities of GA, namely inhibition of HMGB1 and formation of hydrogels, we here explored the physicochemical and biological properties of GA-based hydrogels as bioactive wound dressings. We performed rheological studies, combined with cryogenic scanning electron microscopy (cryoSEM) and small angle X-ray scattering (SAXS) to elucidate the structure, and proved that topical application of GA-based hydrogels is a potent therapeutic strategy to accelerate cutaneous wound healing responses, outperforming commercially available hydrogels and avoiding the need for additional gelling agents.

## 2. Experimental Section

### 2.1. Materials

Glycyrrhizin ammonium salt 98% was obtained from ACROS. 3M Tegaderm wound filler was purchased from the pharmacy.

### 2.2. Procedures/Data

#### 2.2.1. Methods

The GA (5%) was prepared by adding 21 mg GA to 400 mL deionized water heated at 80 °C while stirring. When the GA powder was dissolved, heating was stopped, and hydrogels were formed while cooling. Similarly, this was performed on the 10 wt.% (44 mg/400 mL deionized water). For rheological and SAXS experiments, the hydrogels were used as is, while for wound healing experiments, the hydrogels were sterilized by autoclaving.

All rheological measurements were performed on the Anton Paar MCR501, stress-controlled rheometer with a torque range between 0.01 µNm and 300 mNm and temperature range of −160 °C and 1000 °C. The chosen geometry setup was a Couette cell consisting of a bob (height 100 mm and diameter of 26.66 mm) and cup (volume 19.35 mL). The sample was liquefied prior to the measurement and was poured into the cup, while the bob was present in the cup, avoiding air bubbles.

The CryoSEM of samples prepared or kept at 25 °C were conducted as follows: the samples were cooled down in nitrogen slush at atmospheric pressure, freeze-fractured at −180 °C, etched for 60 s at −98 °C, broken by knife, sputtered with platinum in the GATAN Alto 2500 Cryo preparation chamber, and transferred to the cryo-SEM. Similar experiments were performed with samples of 37 °C, 40 °C, 48 °C, and 50 °C.

The SAXS samples were prepared by heating the hydrogel at 80 °C. The liquefied samples were transferred to a 1 mm borosilicate glass capillary with a syringe and needle and sealed with a hot glue gun. X-ray scattering experiments were performed on a Xeuss 2.0C laboratory beamline (Xenocs, Sassenage, France) using copper Kα (λ = 1.54189 Å, 50 kV, 0.6 mA) radiation. The X-ray source used collimated optics with a divergence of 0.4 mrad and a beam size of approximately 0.25 mm^2^ at the sample position. Sample capillaries were mounted on a Linkam HFSX-350 temperature control stage. Scattering patterns were collected on a DECTRIS EIGER 1M detector and corrected to absolute intensity by measurement of the transmitted beam on the detector before and after sample measurement and normalization to exposure time. The entire beam path (source to detector) was evacuated to high vacuum during measurements to reduce incoherent scattering contributions due to air. SAXS patterns were collected at a sample to detector distance of ~1190 mm (actual value used during data processing). The sample to detector distance and scattering vector were calibrated by measurement of silver(I) behenate under identical conditions. Measurements were performed using SPEC (Gainesville, VA, USA, Certified Scientific Software, https://www.spec.org/, accessed on 26 April 2022) with Linkam T96 control macros supplied by Xenocs. Prior to the first measurements, the blank scattering (scattering of a capillary filled with distilled water) was recorded for 6 × 600 s. This contribution was subtracted (after azimuthal integration) from the scattering intensities of the samples to obtain the scattering contribution of the pure hydrogels. For the SAXS temperature experiments, the samples were first heated to 80 °C and cooled down to the measurement temperature. When reaching this new temperature, the sample was equilibrated for 30 min ensuring homogenous temperatures throughout the sample. At each of the indicated temperatures, 6 measurements of 600 s were performed. The resulting measurements were summed to improve signal to noise ratio.

Differential scanning calorimetry (DSC experiments) were performed using a DSC Q2000 (TA Instruments, Brussels, Belgium) equipped with an autosampler. Approximately 10–20 mg of hydrogel was sealed in a hermetic aluminum pan (TA Instruments). The following method was used: equilibration at 10 °C, followed by heating at 5 °C/min to 80 °C thereby melting the sample, subsequent cooling at 5 °C/ min to 10 °C. The heating/cooling cycle was repeated. The gelation temperature (T_gel_) was determined by taking the maximum of the gelation peak.

#### 2.2.2. Mice

The following mouse lines were used: C57Bl/6J and B6.BKS(D)-Leprdb/J (Lepr^db/db^ mice) (Jackson Laboratories, Bar Harbor, ME, USA). All animal procedures were conducted in accordance with European, national, and institutional guidelines (EU Directive 2010/63/EU for Animal Experiments) and protocols were subject to local ethical approval of VIB-Ghent University.

#### 2.2.3. Skin Wound Healing Studies

C57Bl/6J and Lepr^db/db^ mice were subjected to full-thickness skin wounding as previously described [9]. Mice were housed in individually ventilated cages at the VIB Center for Inflammation Research, in a specific pathogen-free animal facility. Back skin of 8-week-old female mice was shaved, and wounds were made using an 8 mm punch biopsy needle (Stiefel Instruments, London, UK) under analgesia and general anesthesia. Wound sizes were measured every other day using electronic calipers. Mice were topically treated with 200 μL of the respective hydrogel every other day, starting at the day of wounding. Wound healing results shown are indicative of three independent experiments. No animals were excluded from any experiment.

#### 2.2.4. Skin Histology and Immunofluorescence

Wounded skin regions were isolated and fixed using 4% paraformaldehyde overnight or embedded in Optimal Cutting Temperature (OCT) compound for frozen sections. Following dehydration steps, samples were embedded in paraffin and sectioned at 10 μm thickness. Dewaxed paraffin wound sections were stained with hematoxylin and eosin stains and imaged using a slide scanner (Zeiss AxioScan). Frozen tissue blocks were sectioned (10 μm) with a cryostat and fixed in 4% paraformaldehyde for 1 h. After fixation, tissue was blocked in buffer containing 5% rat serum, 1% fish gelatin, and 3% Triton X-100 in PBS for 2 h at room temperature before incubation with primary antibody (1:300 Itgα5, clone 5H10-27) at 4 °C overnight.

#### 2.2.5. ELISA

Blood was collected by cardiac puncture and left to clot for 2 h, after which blood was centrifuged and serum was collected. HMGB1 levels were quantified in 50 μL serum by ELISA (Tecan ST51011) according to the manufacturer’s instructions.

#### 2.2.6. Statistical Analysis

Statistical significance was determined using GraphPad Prism 9, using two-way ANOVA with multiple comparisons, One-way ANOVA or Brown-Forsythe and Welch ANOVA testing. * *p* < 0.05, ** *p* < 0.01, *** *p* < 0.001, **** *p* < 0.0001 were considered significant.

## 3. Results and Discussion

### 3.1. Rheological Properties of GA-Based Hydrogels as a Function of GA Concentration

To gain insight into the viscoelastic properties of hydrogels containing either 5% GA (GA (5)) or 10% (GA (10)), we performed rheology studies using a Couette cell (bob and cup), as other geometries gave rise to a wall slip, impeding reproducibility. Both hydrogels were investigated at 20 °C (room temperature) and 37 °C (application temperature). Rheological characteristics were investigated by oscillatory tests (strain (γ), frequency (ω), and temperature sweeps) and rotational tests (shear stress (τ) sweep, step shear rate and hysteresis tests). The main parameters that were evaluated were G’ (storage modulus), representing the solid, elastic, character of the gel, and G” (loss modulus), representing the liquid, viscous, character of the gel.

**Figure 1:**
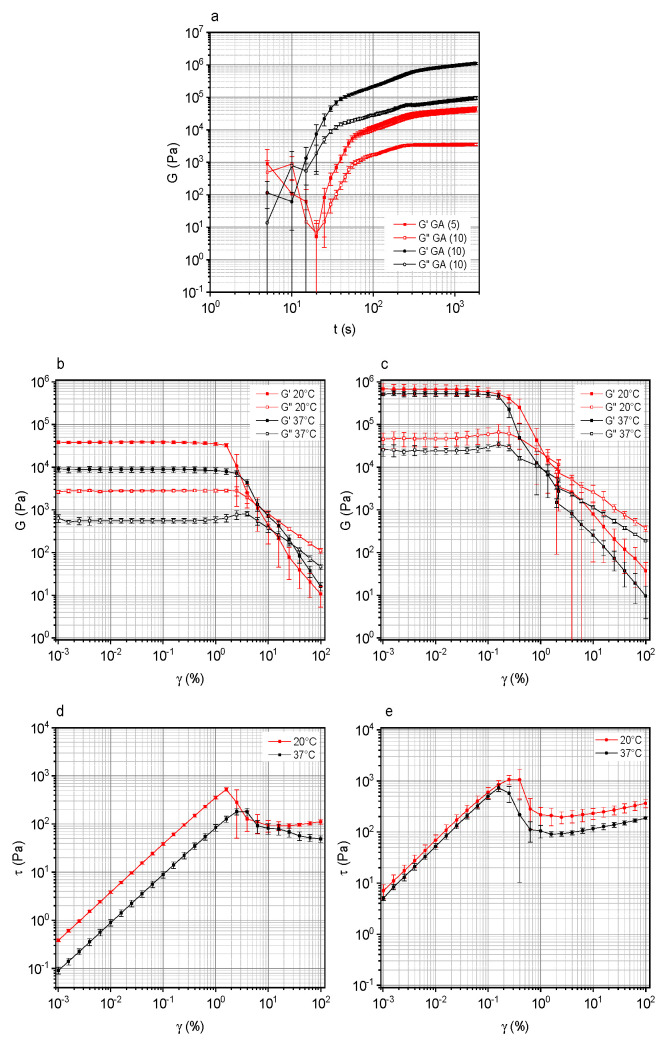
(**a**). Time (t) sweep of GA (5) (red) and GA (10) (black) with a Couette cell. (ω = 10 rad s^−1^, γ_0_ = 0.01%) at 20 °C Graph depicts the storage (G’) and loss (G’’) moduli versus time. (**b**) Strain sweep of GA (5) hydrogel at 20 °C (red) and 37 °C (black). (ω = 10 rad s^−1^, γ_0_ = 0.001–100%) (**c**) Strain sweep of GA (10) hydrogel at 20 °C (red) and 37 °C (black). (ω = 10 rad s^−1^, γ_0_ = 0.001–100%). Graph depicts the storage (G’) and loss (G’’) moduli versus strain (γ) (Pa). (**d**) Shear stress (τ) vs. strain (γ) of GA (5) hydrogel at 20 °C and 37 °C. (ω = 10 rad s^−1^, γ_0_ = 0.001–100%). (**e**) Shear stress (τ) vs. strain (γ) of GA (10) hydrogel at 20 °C and 37 °C. (ω = 10 rad s^−1^, γ_0_ = 0.001–100%).

First, the gelation time of the GA hydrogel was monitored to quantify the speed of structure and hydrogel formation. The sample was heated above its gelation temperature (~80 °C) to reach its sol phase, this gel-sol process is the transformation of a colloidal network (gel phase) into colloidal suspension (sol phase). Thereafter, the liquid sample was added to a temperature-controlled cup (20 °C) with the bob placed in the cup. A time sweep experiment was started prior to the addition of the hydrogel. The gelation time was defined as the time at which both moduli were within 95% percent of the final plateau (Figure 1a). The hydrogel started as a liquid and within 200 s, the storage modulus (G’) rose above 10^4^ Pa for GA (5) and 10^5^ Pa for GA (10). After 200 s, the gelation curve still increased, albeit at a slower rate, proving that initial network formation takes place rapidly while the system needs longer to reach its final strength (1300 s for GA (5) and 1500 s for GA (10)). The GA (10) network formation was found to be faster than GA (5), however, the GA (10) network takes longer to reach its final strength. Both these effects can be attributed to the higher GA concentration.

Next, the deformation of the hydrogels was assessed by strain sweep analysis. The storage modulus (G’) and the loss modulus (G”) versus strain plots display two zones: the first zone is the linear viscoelastic domain, where there is no effect of strain on either of the moduli, while the second zone represents hydrogel network breakdown, where both moduli decrease (Figure 1b,c). For both concentrations, the storage modulus (G’) was larger than the loss modulus (G”) in the linear viscoelastic domain, indicating that they were bona fide gels. Both GA (5) and GA (10) had a large linear viscoelastic domain, with the GA (10) linear domain being smaller; the moduli began to deviate at a strain of 1% vs. 0.06% for GA (5) at 20 °C. Prior to gel breakdown, a small increase in G” was observed, caused by increased formation of microcracks [45,46]. This so-called weak strain and overshoot is common for heavily extended structures showing resistance to deformation [47]. When the corresponding strain becomes too high, these extended structures align and samples flow, indicated by G’ becoming less than G”. The G’-G” cross-over point was concentration dependent occurring at a strain of 6.3% for GA (5) and 1.4% for GA (10). The breakdown of the GA (10) hydrogel at lower strains indicates a more brittle hydrogel.

The strength of the GA network depends on temperature and the glycyrrhizin concentration. The thermoresponsive effect on the GA (5) resulted in a decrease in storage modulus G’ in the linear viscoelastic domain from 38 ± 2.4 kPa at 20 °C to 8.9 ± 1.4 kPa at 37 °C. There was only a minor temperature effect on the GA (10) hydrogel with the difference between both moduli being less significant: 672 ± 20 kPa (20 °C) and 522 ± 68 kPa (37 °C). These results were confirmed by differential scanning calorimetry (DSC) (Supplementary Figure S1).

**Figure 2:**
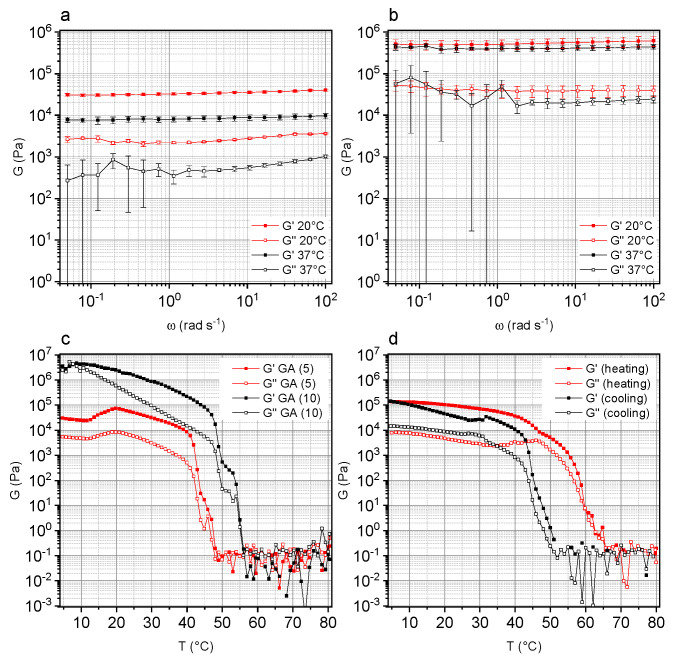
(**a**) Frequency (ω) sweep of GA (5) hydrogel with a Couette cell (ω = 0.05–100 rad s^−1^, γ_0_ = 0.01%) at 20 °C (red) and 37 °C (black). (**b**) Frequency sweep of GA (10) hydrogel (ω = 0.05–100 rad s^−1^, γ_0_ = 0.01%) at 20 °C (red) and 37 °C (black) (**c**) Temperature (T) sweep of GA (5) and GA (10) hydrogel with a Couette cell (ω = 10 rad s^−1^, γ_0_ = 0.01%) during cooling from 80 °C to 5 °C with a cooling rate 0.01 °C/s. (**d**) Temperature (T) Sweep of a GA (5) hydrogel (ω = 10 rad s^−1^, γ_0_ = 0.01%) during heating and cooling.

For its application as a wound healing gel, the flow behavior will influence how the hydrogel is to be applied to the wound with the yield stress—the minimum shear stress needed to initiate or terminate flow—being a critical parameter. The yield stress is the maximum shear stress obtained during the strain sweep (Figure 1d,e). GA (5) had a yield stress of 523 ± 44 Pa at 20 °C, and 181 ± 27 Pa at 37 °C. For GA (10), the yield stress was higher, with 1038 ± 610 Pa at 20 °C and 736 ± 110 Pa at 37 °C. Next, the time dependent behavior of the hydrogels was investigated by applying a constant strain in the linear domain of the strain sweep (0.01%) and varying the frequency. Frequency sweeps were conducted at 20 °C and 37 °C and showed a stable mechanical strength for both gels over time (Figure 2 a,b). The strength of the GA (5) hydrogel dropped half a decade as both storage and loss modulus decreased comparable to the strain sweep at 37 °C. This decrease was not visible for the GA (10) hydrogel. It is remarkable that the GA physical hydrogels show such high values for both G’ and G”, indicating the strength of these gels.

To evaluate the strength of the hydrogel as a function of temperature and to determine the sol-gel transition (T_sol-gel_), a temperature sweep (0.01 °C/s) was performed. The temperature range of the thermoresponsive behavior of the GA hydrogels was thus determined (Figure 2c,d). The liquid hydrogel was cooled down while both moduli were monitored. During cooling, the steep rise in moduli indicated that the fibrillar network was formed, while during heating a decrease in the moduli was observed corresponding to network breakdown. The transition between the gel and the sol phase is a measure for the applicability of the hydrogel at body temperature. Figure 2b shows both the moduli of GA (5) during cooling (black curves) and heating (red curves). At elevated temperatures (>60 °C) the moduli are below 1 Pa, indicating no resistance to deformation and a dominant liquid-like behavior. During cooling, the sample remained liquid until 50 °C (Figure 2d, black curves). Further cooling resulted in a rise of both moduli while G’ > G” showed the solid behavior of the hydrogel. The heating curve (red curve, Figure 2d) was slightly different with the hydrogel network remaining stable over a larger temperature domain. Hence, network breakdown appears to be slower than network formation. Similar hysteresis was observed earlier on a GA hydrogel containing 9.2 % [41]. The slower breakdown was also translated into a significantly higher de-gelation temperature (60 °C) compared to the gelation temperature (40 °C). The moduli during cooling and heating were in the same range of the frequency sweep, indicating that this test was reliable. This also shows that the GA (5) can be applied as a gel on mammalian skin. This hysteresis was independent on GA concentration as it was also observed for GA (10) (Supplementary Figure S2).

GA (5) and GA (10) have similar behavior while showing different heating and cooling curves with the curves shifted towards higher temperatures for GA (10) (Figure 2c,d). During cooling, there was a steep increase in both G’ and G”, indicating hydrogel network formation and corresponding structure build-up. This sol-gel transition is induced by the presence of rodlike micelles [48] (low values for G’ and G”) at elevated temperatures. As temperature decreases, these micelles fuse into larger constructs (G’ and G” with higher values). The T_gel-sol_ for GA (5) was 40 °C while T_gel-sol_ for GA (10) was 48 °C, while heating (Supplementary Figure S2). Differential scanning calorimetry showed similar transition temperatures (Supplementary Figure S1).

### 3.2. Structural study

**Figure 3:**
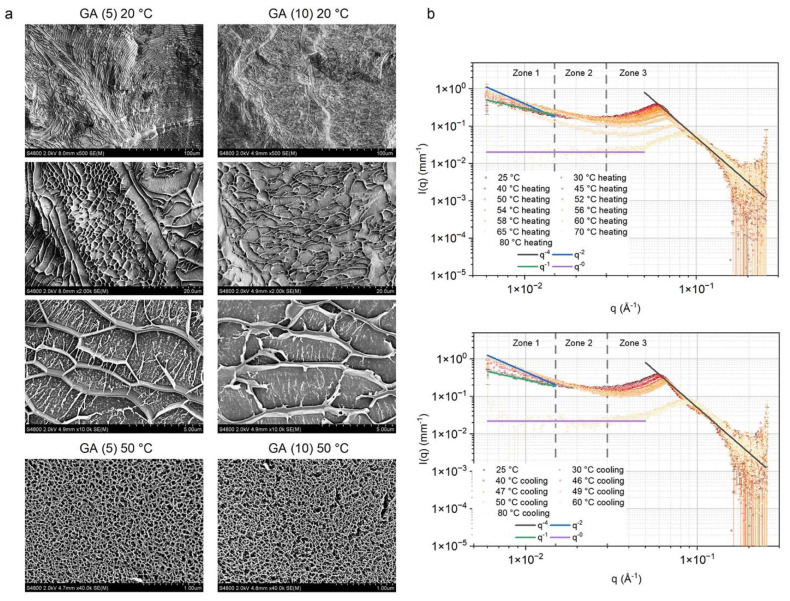
(**a**) CryoSEM micrographs of GA (5) and GA (10) at 20 °C and 50 °C. (**b**) Temperature dependent Scatter profiles of GA (10) during cooling (bottom) and during heating (top).

The rheological study showed large differences in strength between the sol and the gel phase of the hydrogels. As the hydrogels consisted solely of water and GA, these changes in properties are caused by differences in the self-assembly of the GA molecules. This self-assembly is based on the crystalline structure of GA (Supplementary Figure S3), forming individually right-handed fibrils at lower concentrations [42]. These isolated fibers fail to explain the observed strength of the hydrogel, especially at room temperature. To get an insight into the mesoscale dimensions (micrometer to nanometer) of the glycyrrhizin-based hydrogels, we performed cryoSEM imaging (Figure 3a) and SAXS measurements (Figure 3b, Figures S4 and S5) on both the GA (5) and GA (10) gels. CryoSEM imaging showed leaf-like wall textures, such as networks of lamellar bricks with cavities. Such cavities were previously reported, however, not in such detail [49]. We determined the distance between two lamellar-like walls forming the cavity to be predominantly in the range of 1.47 μm to 3.30 µm, while the lengths of the walls were up to several hundred micrometers.

The wall arrangement appears inhomogeneous over the whole structure but contains regions of self-similarity in spots smaller than 20 µm (Figure 3a middle). The walls resemble long stretched combs and contain multiple branching and crosslink points, which explains the strength of the hydrogel network. The big comb-like walls have a thickness of 100–180 nm for GA (5) and a height/width up to 500 nm. For GA (10), we observed a wall thickness of 100–250 nm, an average wall height of 250 nm, and a higher number of crosslinking points. These differences explain the significantly higher strength of GA (10) compared to GA (5).

Cryo-SEM images at the sol state (50 °C) showed a network structure consisting of an irregular fibrillar structure with an average fibril size of 100 nm (Figure 3a lower panels). The observed structures in the sol state show that the comb like superstructure had vanished and disintegrated into irregular fibrillar structures, indicating that the more regular larger fibril structures are responsible for the hydrogel strength. To complement the rheological studies, temperature-dependent SAXS measurements were carried out (Figure 3b and Figure S4). Since the scattering vector q is highly sensitive to different dimensions of the internal GA morphology, the scattering intensities I(q) are discussed in three zones: (a) zone 1 (q < 0.015 Å^−1^), governed by characteristic features in the mesoscale, (b) zone 2 (0.015 Å^−1^ < q < 0.03 Å^−1^), sensitive to (sub-) structures with (partial) sizes in the range of ca. 20–50 nm, and (c) zone 3 (0.3 Å^−1^ < q), which gives information about GA features smaller than 20 nm. The 1D-SAXS data of the limiting cases of GA gel at 25 °C and sol at 80 °C are indicated in Supplementary Figure S4.

The phase transition between gel and sol by temperature dependent SAXS measurements (Figure 3b and Figure S5) revealed reversible hysteresis. The morphological changes seemed to be similar during heating and cooling, but the phase transition was more abrupt during cooling, indicating different kinetics. For GA (10), the transition appeared between 60 °C and 70 °C during heating and between 60 °C to 55 °C during cooling. The corresponding values for GA (5) were 55 °C to 60 °C (heating) and 50 °C to 45 °C (cooling). In zone 1 a continuous change from q^−1^ to q^−2^ with increasing temperature was observed in the gel state (Figure 3b) red and yellow line (zone 1), indicating scattering dominance of mesoscale 2D structures. At the same time, the correlation peak in zone 3 shifted to larger q values indicating a slight decrease in the size of the small (micellar) building blocks. As expected, a significant smaller partial unit dominated scattering in the sol state.

**Figure 4:**
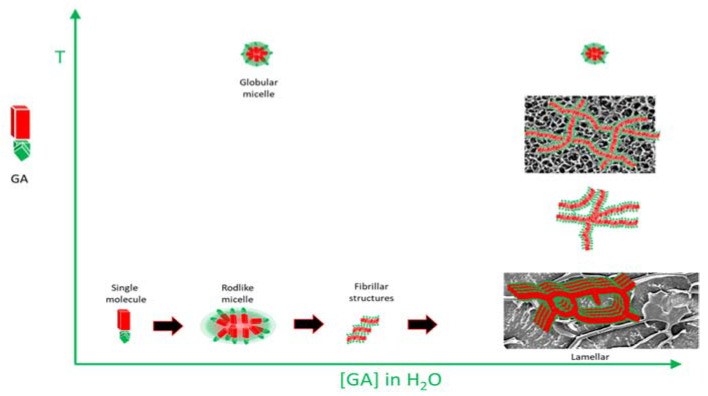
Structure formations of GA according to the temperature and concentration. Self-assembly of the GA molecules into micelles, fibrillar structures, and subsequently into lamellar structures. These thermoresponsive structures then disassemble into fibrillar structures and micelles upon heating.

The crystal structure of solid GA has previously been reported in the literature and the authors hinted that the crystal structure might be formed in solution [50,51,52,53]. GA forms alternating hydrophilic and hydrophobic areas in the crystal and the authors suggested that a similar structure might be formed in concentrated solutions [54]. These assemblies can be considered as bilayers consisting of a hydrophilic interior formed by a two-dimensional sugar platform and aglycon fragments protruding from its surface on both sides. Dependent on the concentration and the temperature either water or cationic molecules may penetrate neighboring hydrophilic areas. This morphology allows for crosslinking and the formation of more complex structures like bilayers with columns and crosses or even morphologies like networks or sponges (Figure 4).

### 3.3. GA-Based Hydrogels Accelerate Wound Closure of Murine Skin Wounds in Normoglycemic and Diabetic Conditions

**Figure 5:**
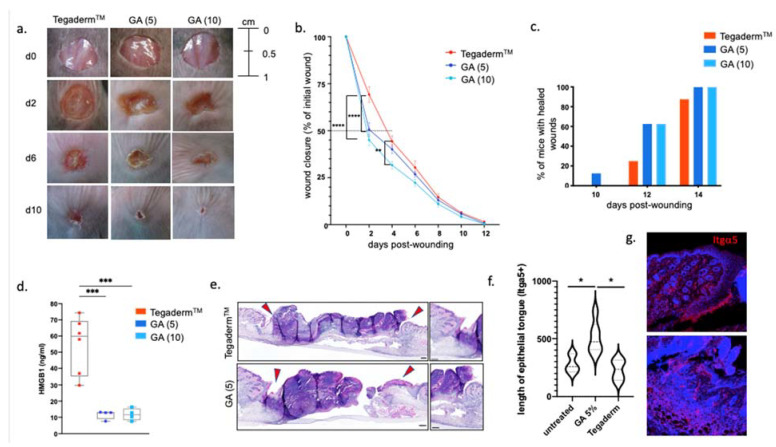
GA-based hydrogels accelerate wound healing in normoglycemic mice. (**a**) Representative pictures of wounds on the back skin of normoglycemic C57BL/6J mice at different days post-wounding treated with different hydrogels. Scale bar: 1 cm. (**b**) Wound closure kinetics of normoglycemic mice treated with GA (5%), GA (10%) or Tegaderm^TM^ wound filler (*n* = 10 per condition). Wound size is expressed as percentage of initial wound size (** *p* < 0.01; **** *p* < 0.0001; Two-way ANOVA with multiple comparisons). Data represent means ± SEM. (**c**) Percentage of mice with fully closed wounds at different days post-wounding. (**d**) Serum levels of HMGB1 in wounded mice treated with different hydrogels at day 5 post-wounding as assessed by ELISA (One-way ANOVA; *** *p* < 0.001; *n* ≥ 4 per condition). (**e**) Hematoxylin-eosin stained wound sections of differently treated wounds at day 5 post-wounding. Scale bar: 200 mm. Red arrowheads indicate the wound edge. (**f**) Quantification of the length of the epidermal tongue as measured by Itgα5 positive staining (* *p* < 0.05; Brown-Forsythe and Welsch ANOVA test). (**g**) Immunofluorescent images of Itgα5 staining of wound sections at day 3 post-wounding treated with GA (5%) or or Tegaderm^TM^ wound filler.

In order to test the regenerative properties of GA-based hydrogels in vivo, we wounded C57Bl/6J mice with an 8 mm full-thickness punch biopsy of the skin and repetitively applied GA-based hydrogels. Wound closure was monitored every other day for 14 days followed by topical treatment with the respective hydrogel. GA (5) and GA (10) hydrogels were tested alongside Tegaderm^TM^ hydrogel (Tegaderm^TM^ Wound Filler, 3M, Saint Paul, MN, USA), a commercial hydrogel used for the management of diabetic ulcers. Wounds treated with GA (5) or GA (10) hydrogels closed markedly faster than Tegaderm^TM^-treated wounds (Figure 5a,b). Wounds of GA (5) or GA (10) hydrogel-treated mice reached 50% wound closure at day 2 post-wounding, nearly two days earlier than Tegaderm^TM^-treated wounds (Figure 5a,b). This is in agreement with the previously reported accelerated wound healing during the inflammatory stage of injury repair in mice lacking HMGB1 in keratinocytes relative to controls [10]. At day 12 post-wounding, 60% of the wounds treated with GA (5) or GA (10) were fully re-epithelialized compared to 20% of wounds treated with Tegaderm^TM^ hydrogel (Figure 5c). Efficient blocking of HMGB1secretion by topical application of GA-based hydrogels was confirmed in serum of mice at day 5 post-wounding (Figure 5d). Histological analysis of the differently treated wounds at day 4 post-wounding revealed a shorter eschar in GA (5) hydrogel-treated wounds and longer epithelial tongue, indicating faster migration of keratinocytes in GA-based hydrogel-treated wounds relative to Tegaderm^TM^-treated wounds (Figure 5e). The increased keratinocyte migration rate in wounds treated with GA-based hydrogels was confirmed by quantifying the length of the epidermal tongue, as assessed by Integrin-α5 (Itgα5) staining (Figure 5f,g).

**Figure 6:**
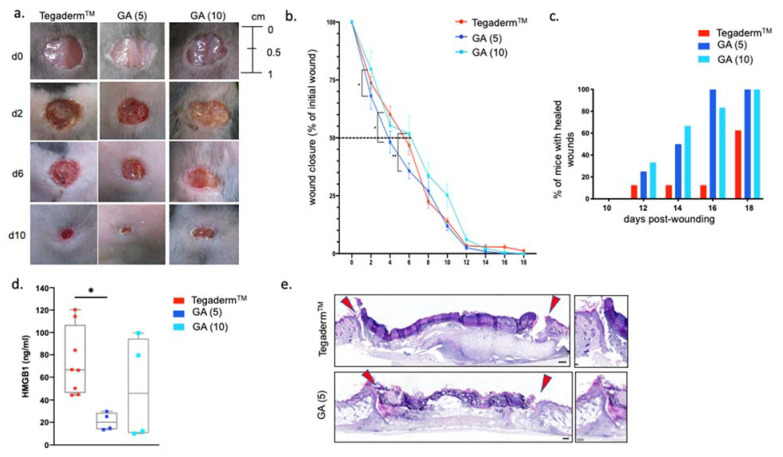
GA-based hydrogels accelerate wound healing in diabetic mice (**a**) Representative pictures of wounds on the back skin of diabetic Lepr^db/db^ mice at different days post-wounding treated with different hydrogels. Scale bar: 1 cm. (**b**) Wound closure kinetics of diabetic mice treated with GA (5%), GA (10%) or Tegaderm^TM^ wound filler (*n* = 10 per condition). Wound size is expressed as percentage of initial wound size (* *p* < 0.05; ** *p* < 0.01; Two-way ANOVA with multiple comparisons). Data represent means ± SEM. (**c**) Percentage of mice with fully closed wounds at different days post-wounding. (**d**) Serum levels of HMGB1 in wounded diabetic mice treated with different hydrogels at day 5 post-wounding as assessed by ELISA (One-way ANOVA; * *p* = 0.0405; *n* ≥ 4 per condition). (**e**) Hematoxylin-eosin stained wound sections of differently treated wounds at day 5 post-wounding. Scale bar: 200 mm. Wound edges are indicated by red arrowhead.

Next, we aimed to investigate whether our GA-based hydrogels could also accelerate regenerative skin responses in a diabetic context. For this, we wounded Lepr^db/db^ mice, representing a well-established mouse model for type 2 diabetes disease, expressing a defective leptin receptor and exhibiting impaired wound healing [55,56]. Full-thickness wounding was performed in Lepr^db/db^ mice and wounds were measured every other day, followed by treatment with GA (5), GA (10), or Tegaderm^TM^ hydrogel. In the initial stages of wound healing, diabetic wound closure was significantly accelerated in GA (5) hydrogel-treated wounds relative to GA (10) or Tegaderm^TM^ hydrogel-treated wounds (Figure 6a,b). Wounds in GA (5) hydrogel-treated diabetic mice reached 50% of wound closure at day 4 post-wounding, nearly two days earlier than Tegaderm^TM^-treated wounds (Figure 6a,b). At day 16 post-wounding, nearly all wounds treated with GA (5) or GA (10) were fully re-epithelialized compared to 10% of wounds treated with Tegaderm^TM^ hydrogel (Figure 6c). Reduced HMGB1 levels were observed in serum of mice treated with GA (5) hydrogels relative to Tegaderm^TM^-treated mice (Figure 6d). Histological analysis of the differently treated wounds at day 6 post-wounding revealed less granulation tissue and longer epithelial tongues in GA (5) hydrogel-treated wounds relative to Tegaderm^TM^-treated wounds (Figure 6e). In conclusion, topical treatment of wounds with GA (5) hydrogels accelerated cutaneous wound closure compared to treatment with Tegaderm^TM^ hydrogel in both normoglycemic and diabetic skin.

## 4. Conclusions

The structural and rheological properties of a hydrogel containing 5% or 10% GA were investigated showing stable and strong physical hydrogels with thermoresponsive behavior. Both strength, elasticity, and T_sol-gel_ were affected by the GA concentration of these gels. The gel forming structure was studied by means of CryoSEM and SAXS showing large macrostructures containing fibrils. Cutaneous wound healing studies on normoglycemic and diabetic mice showed a decrease in healing time and improved healing characteristics when mice were treated with GA-based hydrogels relative to no treatment or treatment with a commercial hydrogel.

## Data Availability

Data will be made available upon request.

## Supplementary Materials

The following supporting information can be downloaded at: https://www.mdpi.com/article/10.3390/pharmaceutics15010027/s1, Figure S1: DSC cooling and heating cycles of GA (5) and GA (10) hydrogels. Figure S2: Temperature sweep of a GA (10) hydrogel during cooling and subsequent heating. Figure S3: Crystalline structure of GA. Figure S4: SAXS intensities of the GA (5) and GA (10) hydrogel. Figure S5: Temperature dependent scatter profiles of GA (5) during cooling (a) and during heating (b).
