# Supplementary material for: Glycyrrhizin-Based Hydrogels Accelerate Wound Healing of Normoglycemic and Diabetic Mouse Skin
Source: Pharmaceutics. 2022 Dec 21;15(1):27. doi: 10.3390/pharmaceutics15010027 (PMC9861362; doi:10.3390/pharmaceutics15010027)
Supplement: Supplementary file 1 [file pharmaceutics-15-00027-s001.zip › pharmaceutics-2063877-supplementary.pdf]

Supplementary Information

Glycyrrhizin-Based Hydrogels Accelerate Wound Healing of Normoglycemic and Diabetic Mouse Skin

Maarten A. Mees [1], Fleur Boone [2,3], Thomas Bouwen [1], Frederik Vanaerschot [1], Charlotte Titeca [1], Hanna-Kaisa Vikkula [2,3], Leen Catrysse [2,3], Anja Vananroye [4], Erin Koos [4], Stelios Alexandris [1], Sabine Rosenfeldt [5], Samuel Eyley [1], Joachim Koetz [6], Geert van Loo [2,3], Wim Thielemans [1,*] and Esther Hoste [2,3,*]

[1] Sustainable Materials Lab, Department of Chemical Engineering, Campus Kulak Kortrijk, KU Leuven, Etienne Sabbelaan 53, 8500 Kortrijk, Belgium
[2] VIB Center for Inflammation Research, Technologiepark 71, 9052 Ghent, Belgium
[3] Department of Biomedical Molecular Biology, Ghent University, Technologiepark 71, 9052 Ghent, Belgium
[4] Chemical Engineering Department, Soft Matter, Theology and Technology, KU Leuven, 3000 Leuven, Belgium
[5] Physical Chemistry and Bavarian Polymer Institute (BPI), University of Bayreuth, Universitätsstraße 30, 95447 Bayreuth, Germany
[6] Institut für Chemie, Universität Potsdam, Karl-Liebknecht-Straße 24-25, 14476 Potsdam, Germany
* Correspondence: wim.thielemans@kuleuven.be (W.T.); esther.hoste@irc.vib-ugent.be (E.H.); Tel.: +32-(0)56-24-61-71 (W.T.); +32-(0)9331-37-63 (E.H.)

Supplementary Figures and Results

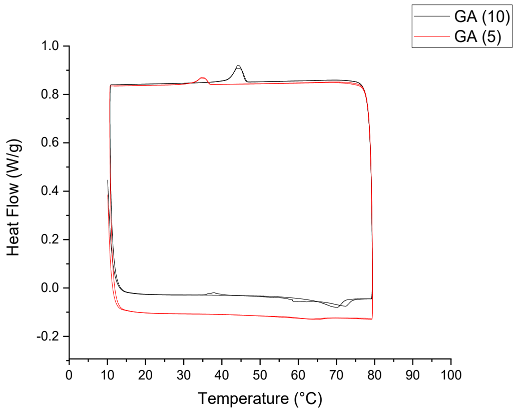

Supplementary Figure S1. DSC cooling (top curves) and heating (bottom curves) cycles of GA(5) and GA(10) hydrogels from 10-80 °C with heating/cooling rate 5°C/min.

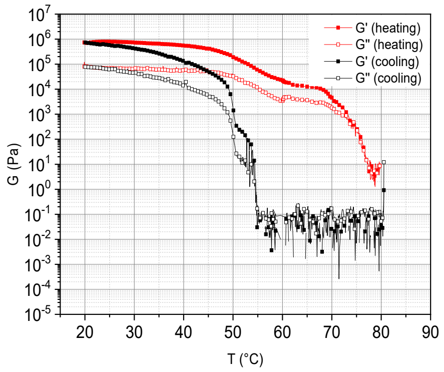

Supplementary Figure S2. Temperature (T) Sweep of a GA (10) hydrogel with a Couette cell (ω = 10 rad s^{-1},γ_0 = 0.01%) during cooling from 80°C to 5°C with a cooling rate 0.01°C s^{-1} and subsequent heating at the same rate.

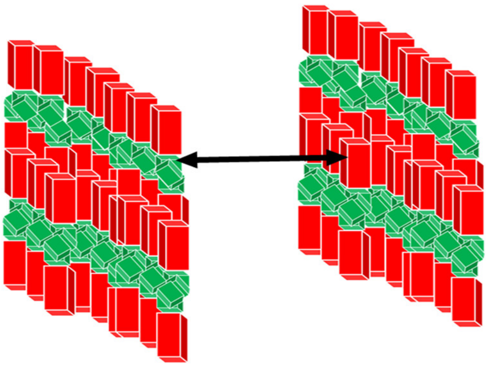

Supplementary Figure S3. Crystalline structure of GA, which is a plane passing through the carbon atoms attached to the carboxylic groups which divides the GA molecule in such a way that these groups are all located on one side whereas the carbohydrate hydroxyl groups are found on the opposite plane side. In this way GA forms alternating hydrophilic and hydrophobic area in the crystal. The unit cell of a corresponding crystal is 10.66 Å x 11.12 Å x 51.81 Å.51.

Results and discussion

All characteristic features of GA, i.e. the self-assembly structure as well as the underlining building blocks, contribute to the SAXS pattern, but manifest themselves in different scattering regions (zones in Supplementary Figure 1).

At 80°C the intensities of GA (5) and GA (10) appear similar, with differences related to the concentration of GA. In zone 1, the device limitation and the resulting poor statistics hinders a profound analysis. Most likely, the residuals of a porous network with mesoscale dimensions were observed exhibiting a power law of ca. q^{-3} (Supplementary Figure 1, pink straight line in zone 1). This, however, does not preclude the existence of other (sub)structures. Using the Guinier approximation, we determined a radius of gyration of $R_g \approx 1.2$ nm for subunits seen in zone 2-3. The subunit may be a single GA molecule or small GA micelles. Since the dimensions of a GA molecule are approximately (1.1 nm x 1.2 nm x 2.6 nm) [53] corresponding to an estimated R_g around 0.8 nm, the observed structure is most likely a micellar self-assembled structure. The weak correlation peak at q = 0.09 $Å^{-1}$ (d ≈ 7 nm, corresponding roughly two times the longest dimension of a GA molecule), in zone 3 corroborates this conclusion. Thus, GA molecules appear to assemble in pairs. The existence of GA micelles was already suggested in literature: [41,54] *Matsuoaka*[54] reported rod-like micelles with a radius R = 1.5 - 1.8 nm and a length L = 21 nm for lower concentration GA solutions and at elevated pH (5 - 6). Since this size relates to a radius of gyration around 6 nm (approximation for rod-like micelles $R_g = \sqrt{(R^2/2 + L^2/12)}$; for spheres $R_g = \sqrt{(3/5)}\,R$) we suggest shorter, more globular micelles under our conditions. The scaling law (q^{-4}) in zone 3 (blue line SI figure 1) is attributed to a smooth local surface of micellar GA subunits, which are regarded as building blocks of the network structure.

At 25°C the hydrogels exhibit a q^{-1} scaling in zone 1, which may be explained by a dominant scattering contribution of the 1D morphologies seen in the SEM images (Figure 3 (a) highest magnification). The crossover point from q^{-1} to q^{-0} at the beginning of zone 2 points to a real dimension inside the material of ~40 nm (d = 2π/q approximation), a value which cannot yet be explained with the current data set. Most likely, this size corresponds to a characteristic size of a defined macrostructure inside the gel, which is built by assembly of smaller subunits. In zone 3 the scattering from GA (5) and GA (10) was different. For GA (5) the correlation peak was weak with a maximum at q = 0.04 $Å^{-1}$, whereas it is significantly more pronounced and shifted to q = 0.06 $Å^{-1}$ for GA (10). Consequently, the interaction distances for the GA subunits are d = 16 nm (GA (5)) and d = 10 nm (GA (10)). Furthermore, GA (5) has a higher local surface roughness arising from a more complicated surface architecture reflected by (q^{-3} vs. q^{-4}). This could be explained by a concentration dependent ordering of GA and could explain the higher strength of the GA (10) hydrogel.

In zone 1 (q < 0.015 $Å^{-1}$) the scattering is mainly determined by the mesoscale, e.g., by the contribution of self-assembled structures e.g. porous networks or comb-like structures. Scaling laws are a method to interpretate SAXS data. This analysis technique is based on the definition that a particle is not necessarily the whole particle itself – it can also be a real (sub-) domain inside the particle. In that sense the GA hydrogel can be described in terms of its substructures. 1D (fibrils), 2D (comb-like walls) or 3D (bigger 3D structures or the hydrogel network itself) substructures are particles. They contribute to the scattering intensity I(q) with a characteristic scaling law of q^{-1} for 1D, q^{-2} for 2D and q^{-4} for 3D.

Zone 2 (0.015 $Å^{-1}$ < q < 0.03 $Å^{-1}$) is sensitive to dimensions in the size range of 20 - 50 nm. Power law analysis is restricted by the 1D and 3D size limits, i.e., by q^{-x} with x=1 to 4. This means that interferences leading to q^{-0} must be interpretated in a different way. The first way will assign this partial intensity to the form factor scattering of a single (sub-) particle. This is determined by the radius of gyration (R_g, momentum of inertia) and is reflected in a decaying exponential function (Guinier law, I(q) proportional to $\exp(-(q^2R_g^2)/3)$). When (sub-) units or different particles are closely correlated and aligned in an ordered fashion an additional interference peak (Bragg reflection, shoulder in zone 3) appears, which is sharper with more defined packing. The maximum of this peak indicates the averaged distance (d) between two units and can be approximated by d = 2π/q. In a simple approach the surface of a hydrogel or sol consists of fractal-like arrangement of colloidal spheres. According to Porod, the scattering contribution of a local surface, which can be decomposed into small sub-spheres (here colloidal subunits), is characterized by a power law of q^{-x} at high q. Smooth surfaces show a q^{-4} behavior, whereas rough ones show a lower exponent, for example q^{-3}.

Note, that in zone 3 the scattering patterns of both hydrogels exhibit a slight minimum around q = 0.09 $Å^{-1}$ at 25°C (indicated by arrow), which normally indicates a minimum size dimension inside a single building block. This can be related to spheres with a radius ($4.49/q_{min}$ = R) cross-section radius of cylinders. Unfortunately, the exact calculation of this characteristic dimension needs the assumption of a shape model, therefore we only can estimate this value to be around 4-5 nm. Because the longest dimension of a GA molecule is around 2.6 nm, we can conclude, that the sub-units are still self-assembled structures.

Most likely, the residuals of a porous network with mesoscale dimensions were observed exhibiting a power law of ca. q^{-3} (SI Figure 1, pink straight line in zone 1). This, however, does not preclude the existence of other (sub)structures. Using the Guinier approximation, we determined a radius of gyration of $R_g \approx 1.2$ nm for subunits seen in zone 2-3. The subunit may be a single GA molecule or small GA micelles. Since the dimensions of a GA molecule are approximately (1.1 nm x 1.2 nm x 2.6 nm) [53] corresponding to an estimated R_g around 0.8 nm, the observed structure is most likely a micellar self-assembled structure. The weak correlation peak at q = 0.09 $Å^{-1}$ (d ≈ 7 nm, corresponding roughly two times the longest

dimension of a GA molecule), in zone 3 corroborates this conclusion. Thus, GA molecules appear to assemble in pairs. Furthermore, GA (5) has a higher local surface roughness arising from a more complicated surface architecture reflected by (q-3 vs. q-4). This could be explained by a concentration dependent ordering of GA and could explain the higher strength of the GA (10) hydrogel.

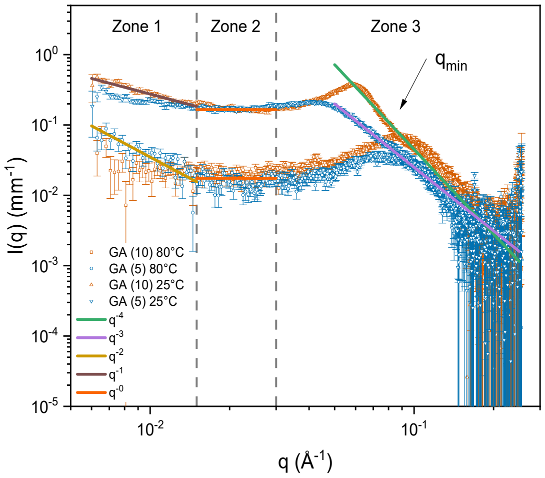

Supplementary Figure S4. SAXS intensities (symbols) of the GA (5) and GA (10) in the limiting cases gel (25°C) and sol (80°C). Additionally, to facilitate interpretation different zones (separated by dotted lines), typical scaling laws (q-x, solid lines) and a form factor minima in the scattering (arrow) are marked.

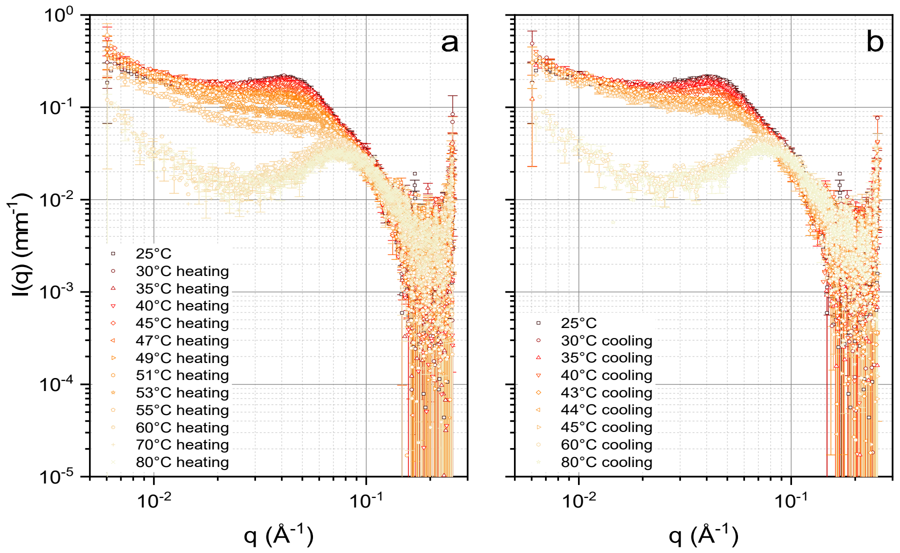

Supplementary Figure S5. Temperature dependent scatter profiles of GA (5) during cooling (a) and during heating (b)
